# Activating transcription factor-2 (ATF2) is a key determinant of resistance to endocrine treatment in an in vitro model of breast cancer

**DOI:** 10.1186/s13058-020-01359-7

**Published:** 2020-11-16

**Authors:** Athina Giannoudis, Mohammed Imad Malki, Bharath Rudraraju, Hisham Mohhamed, Suraj Menon, Triantafillos Liloglou, Simak Ali, Jason S. Carroll, Carlo Palmieri

**Affiliations:** 1 Department of Molecular and Clinical Cancer Medicine, The Institute of Systems, Molecular and Integrative Biology, University of Liverpool, Sherrington Building, Ashton Street, Liverpool, L69 3GE UK; 2 The Clatterbridge Cancer Centre NHS Foundation Trust, Liverpool, UK; 3 Department of Surgery and Cancer, Imperial College London, Faculty of Medicine, London, UK; 4 Cancer Early Detection Advanced Research Center, Oregon Health and Science University, Knight Cancer Institute School of Medicine, Portland, USA; 5 Cancer Research UK, Cambridge Research Institute, University of Cambridge, Cambridge, UK

**Keywords:** ATF2, Tamoxifen, Endocrine resistance, Breast cancer

## Abstract

**Background:**

Activating transcription factor-2 (ATF2), a member of the leucine zipper family of DNA binding proteins, has been implicated as a tumour suppressor in breast cancer. However, its exact role in breast cancer endocrine resistance is still unclear. We have previously shown that silencing of ATF2 leads to a loss in the growth-inhibitory effects of tamoxifen in the oestrogen receptor (ER)-positive, tamoxifen-sensitive MCF7 cell line and highlighted that this multi-faceted transcription factor is key to the effects of tamoxifen in an endocrine sensitive model. In this work, we explored further the in vitro role of ATF2 in defining the resistance to endocrine treatment.

**Materials and methods:**

We knocked down ATF2 in TAMR, LCC2 and LCC9 tamoxifen-resistant breast cancer cell lines as well as the parental tamoxifen sensitive MCF7 cell line and investigated the effects on growth, colony formation and cell migration. We also performed a microarray gene expression profiling (Illumina Human HT12_v4) to explore alterations in gene expression between MCF7 and TAMRs after ATF2 silencing and confirmed gene expression changes by quantitative RT-PCR.

**Results:**

By silencing ATF2, we observed a significant growth reduction of TAMR, LCC2 and LCC9 with no such effect observed with the parental MCF7 cells. ATF2 silencing was also associated with a significant inhibition of TAMR, LCC2 and LCC9 cell migration and colony formation. Interestingly, knockdown of ATF2 enhanced the levels of ER and ER-regulated genes, *TFF1*, *GREB1*, *NCOA3* and *PGR*, in TAMR cells both at RNA and protein levels. Microarray gene expression identified a number of genes known to mediate tamoxifen resistance, to be differentially regulated by ATF2 in TAMR in relation to the parental MCF7 cells. Moreover, differential pathway analysis confirmed enhanced ER activity after ATF2 knockdown in TAMR cells.

**Conclusion:**

These data demonstrate that ATF2 silencing may overcome endocrine resistance and highlights further the dual role of this transcription factor that can mediate endocrine sensitivity and resistance by modulating ER expression and activity.

## Introduction

Globally, breast cancer is a major cause of morbidity and mortality. Oestrogen, which plays a key role in the growth and differentiation of normal breast tissue, is also implicated in the pathogenesis and progression of breast cancer [1], with oestrogen receptor alpha (ERα) being expressed in about 70% of breast cancers. Endocrine therapy [tamoxifen and/or aromatase inhibitors (AI)] has been proven very successful in the clinical setting. However, de novo or acquired resistance to endocrine therapy is a major clinical problem and limits its use [2, 3]. Several studies have identified potential mechanisms of resistance to endocrine therapies such as enhanced growth factor signalling, changes in the expression and/or action of the ER, altered expression of ER co-regulators, micro-RNA or long non-coding RNA interference and DNA methylation [3–7]. In addition, mutations of the *ESR1* gene, encoding the ERα, have been increasingly recognised as an important mechanism of endocrine therapy resistance, mainly to AIs, with a prevalence of around 25% [8, 9]. Transcriptomic profiling of MCF7 tamoxifen-sensitive and its tamoxifen-resistant variant revealed differential expression of genes involved in cell cycle control, transcriptional/translational machinery, *ESR1* regulation, dysfunctional mitochondrial and oxidative phosphorylation and altered metabolism, whereas proteomic analysis of acquired tamoxifen resistance demonstrated downregulated ER-signalling, activation of alternative survival pathways and enhanced cell motility and migration through regulation of the actin cytoskeleton [3, 4, 10–12]. The adaptation of the tumour cell from oestrogen to androgen dependent has been recently demonstrated as another mechanism of resistance to ER-targeted therapies [13]. Understanding the underlying molecular mechanisms that mediate resistance is required to improve the management of endocrine-unresponsive breast cancer, and the development of novel therapeutic strategies in the management of breast cancer.

Activating transcription factor-2 (ATF2), a member of the leucine zipper family of DNA binding proteins, regulates the transcription of various genes, including those involved in apoptosis, cell growth, proliferation, inflammation and DNA damage response [14, 15]. Dependent on its binding partner, ATF2 binds to CRE (cAMP response element) consensus sequences (5-TGACGTCA-3) or to AP-1 (activator protein 1) consensus sequences (5-TGACTCA-3) [14]. ATF2 also possesses an intrinsic histone acetyltransferase activity triggering its own DNA binding effectiveness [16]. However, the exact role of ATF2 in breast cancer is still unclear. ATF2 has been found to increase the transcription of matrix metalloproteinase 13 (MMP13), which may help facilitate breast cancer bone metastasis [17, 18]. In addition, cJun-ATF2 dimers have been shown to lead to the transcription of cyclin A, which increases cell proliferation [19], providing further evidence for a possible oncogenic role for ATF2. Co-culture with malignant epithelial cells in primary human adipose fibroblasts obtained from breast cancer patients increased the levels of phosphorylated ATF2 (pATF2) at the promoter of the aromatase gene responsible for oestrogen synthesis [20]. Furthermore, pATF2 has been shown to facilitate the transcription of MMP2, which increases migration in H-Ras-transformed MCF10A human breast epithelial cells indicating that ATF2 may play a role in breast cancer metastasis [21]. ATF2 also forms a complex with c-Jun and c-Fos that mediates HER2’s induction of cyclooxygenase-2 (COX2), involved in cancer development and metastasis [22]. Several studies reported that v-src causes ATF2 and CREB to bind the CRE/ATF site of the cyclin D1 gene, leading to transcription of cyclin D1 in MCF7 human breast cancer cells [23, 24]. Together, these findings are strongly supportive of a role for ATF2 as an oncogene in breast cancer.

In our previous study, we observed that pATF2 predicts improved disease-free and overall survival in ER-positive breast cancer patients treated with tamoxifen [25]. We also showed that silencing of ATF2 led to a loss in the growth-inhibitory effects of tamoxifen in the ER-positive, tamoxifen-sensitive MCF7 cell line and that tamoxifen treatment caused a dose-dependent phosphorylation of ATF2 within its activation domain, enhancing its transcriptional activity. That work suggested a tumour-suppressive role of ATF2 in ER-positive breast cancer [25]. The apparent dual function of ATF2 has been also observed in skin tumourigenesis, where ATF2 has both oncogenic and tumour-suppressive activities [15, 26, 27]. In the current study, we sought to investigate the in vitro role of ATF2 in acquired resistance to endocrine therapy.

## Materials and methods

### Breast cancer cell lines

MCF7 cells obtained from the Cancer Research UK Cell Services (Clare Hall Laboratories, South Mimms, Herts, UK) were maintained in Dulbecco’s modified Eagle’s medium (DMEM) supplemented with 10% foetal bovine serum (FBS), 5 mM L-glutamine and 1% penicillin/streptomycin (P/S). TAMR cells (MCF7-derived tamoxifen-resistant cell line), a kind gift from Professor RI Nicholson [28] were maintained in DMEM phenol red-free supplemented with 5% charcoal/dextran-stripped FBS (CSS), 1% P/S and 100 nM 4-hydroxytamoxifen (4-OH-Tam) (all the reagents and media were from Sigma-Aldrich Company Ltd., Gillingham, UK). LCC2 (E2-independent, tamoxifen-resistant and ICI 182,780-sensitive subline of the MCF7) and LCC9 (ICI 182,780-resistant MCF7 variant and cross-resistant to tamoxifen) were kind gifts from Professor R. Clarke [29, 30]. LCC2 and LCC9 were cultured similarly to TAMR cells but without tamoxifen.

### ATF2-siRNA transfections, qRT-PCR and immunoblotting

Two small interfering RNA (siRNA) duplexes for ATF2 mRNA depletion (S3492 and S3493) and the non-targeting siRNA (NT-siControl) (Silencer® Negative Control: AM4635) were used in our experiments (Thermofisher Scientific, Paisley, UK). Cells (2.5 × 10^5^ cells/well in 6-well plate) were transfected with 100 nM of each of the ATF2-siRNA duplexes including the NT-siControl using RNAiMAX, according to the manufacturer’s instructions (Thermofisher Scientific, Paisley, UK) and incubated in DMEM phenol red-free medium with 5% CSS. Following overnight incubation, the transfected cells were changed to their standard growth medium for 48 h. RNA was prepared using the Qiagen RNeasy kit (Crawley, UK) and converted to cDNA with the High-Capacity cDNA reverse transcription kit, followed by quantitative real-time PCR (qRT-PCR) using the TaqMan Gene Expression Assays, listed in supplementary Materials and Methods, Table 1 (all from Thermofisher Scientific, Paisley, UK).

Protein extracts and immunoblotting was performed as previously described [25]. Briefly, following ATF2 knockdown, cell lysates were prepared in RIPA buffer (Sigma-Aldrich Company Ltd., Gillingham, UK) supplemented with protease/phosphatase inhibitors (Roche Diagnostics, Burgess Hill, UK). Proteins were separated in 12% SDS-PAGE and probed with primary antibodies at 4 °C overnight. Following secondary horseradish peroxidase-conjugated antibody incubation (Dako UK Ltd., Cambridgeshire, UK), membranes were developed with Super-signal West Pico Chemiluminescent Substrate (VWR International Ltd., Lutterworth, UK). The antibodies were ATF2, p-ATF2, TFF1, ERα, GREB1, PGR, NCOA3, HER2, ERK1/2 and pERK1/2 (Cell signalling, Danvers, MA, USA) and β-actin (Insight Biotechnology, Middlesex, UK), and their details are listed in supplementary Material and Methods, Table 2.

### Cell growth assay

The SulfoRhodamine B (SRB) cell proliferation assay was used for cell growth determination (GeneCopoeia, MD, USA). For ATF2-siRNA and siControl transfections, 10 μl of siRNA-Lipofectamine complexes were added per 3 × 10^3^ cells/well in a 96-well plate with 150 μl of DMEM phenol red-free medium and 5% CSS. Standard growth medium, with/without ligands, was added to the plates the following day. SRB was performed according to the manufacturer’s protocol and absorbance was measured using a Tecan Infinite M200 (TECAN UK Ltd., Reading, UK) plate reader at 492 nm. Results are expressed as the mean ± standard deviation (SD) of triplicate wells from three independent experiments.

### Soft agar colony formation assay

Soft agar gel-I (1% SA-I) was made from 2% low melting temperature agarose (LMA) (Sigma-Aldrich Company Ltd., Gillingham, UK) mixed with an equal amount of standard medium for siRNA-transfected cells, while soft agar gel-II (1% SA-II) was made from 2% LMA mixed with equal amount of DMEM phenol-red free and 5% CSS with 100 nM tamoxifen for siRNA-transfected cells. SA-I and SA-II were plated at 2 ml/well in a 6-well plate and placed at 4 °C to solidify (basement gel layer). Cells were harvested separately, adjusted with SA-I or SA-II to 1 × 10^4^ cells/ml, plated on top of the pre-set basement gel layer and placed at 4 °C for 10 min. Once set, the cells were incubated at 37 °C in a humidified atmosphere (5% CO_2_) for 28 days. After 28 days, cells were fixed and stained with 5% MTT (3-(4,5-Dimethylthiazol-2-yl)-2,5-Diphenyltetrazolium Bromide (Sigma-Aldrich Company Ltd., Gillingham, UK), for 4 h and counted using an Optronix Gel Count (Oxford Optronix, Oxford, UK). The number of colonies for siControl, ATF2-siRNA1 and ATF2-siRNA2 transfected cells was calculated relative to the un-transfected (vehicle) control. Results are expressed as the mean ± SD of triplicate wells from three independent experiments.

### Invasion and scratch-wound assay

To test the invasive and migratory ability of ATF2-silenced cells, ATF2 knockdown was performed as previously described. The invasion assay was performed in Transwell® plates with polycarbonate membrane inserts pre-coated with Matrigel™ (BD Biosciences, Oxford, UK). The cells were seeded onto the upper compartment filled with 100 μl standard culture medium with 2% (v/v) FBS. The lower compartment was filled with 500 μl medium with 10% (v/v) FBS. The invasive cells that crossed the membrane in a 24-h period were fixed, stained with a 1% Crystal Violet Reagent (Sigma-Aldrich Company Ltd., Gillingham, UK). Cell counting was calculated by light microscope for siControl, ATF2-siRNA1 and ATF2-siRNA2 transfected cells and was calculated relative to the un-transfected (vehicle) control. Results are expressed as the mean ± SD of triplicate wells from three independent experiments.

ATF2-silenced cells were also seeded in 6-well plates at a density of 2.5 × 10^5^ cells/well and an artificial gap was created with a yellow pipette tip. The cells were rinsed several times with the appropriate medium to remove dislodged cells and images of living cells were captured at the indicated time points of 0, 12 and 24 h at a magnification of × 4 using an inverted microscope (Nikon Eclipse TE 2000-U). Images were analysed by ImageJ 1.48v software (National Institute of Health, USA) averaging the position of the migrating cells at the wound edges. Results are expressed as the mean ± SD of triplicate wells.

### Gene expression microarray analysis

Microarray analysis was performed to identify genes that were differentially regulated by ATF2 in TAMR cells when compared to the tamoxifen-sensitive MCF7. ATF2 knockdown and RNA extraction were performed as described above. Four biological replicates were prepared for each experimental condition. Gene expression analysis was carried out on Illumina Human HT12 version 4 arrays and deposited to *Gene Expression Omnibus* (GEO) repository (GSE145548). All data analyses were performed on R using Bioconductor packages [31]. Raw intensity data from the array scanner was processed using the BASH and HULK algorithms as implemented in the bead array package [31]. Log2 transformation and quantile normalisation of the data was performed across all sample groups. Differential expression analysis was performed using the limma package [32]. Differentially expressed genes were selected using a *p* value cut-off of < 0.05 after application of false discovery rate (FDR) correction for multiple testing applied globally to correct for multiple contrasts. Differentially expressed genes were analysed by the ‘transcription factor network’ prediction tool EnrichR [33] to identify top driver transcription factors and pathway analysis (KEGG and REACTOME) was performed using the publically available STRING (Functional protein association networks) database v11 (string-db.org).

### DNA methylation

ATF2 knockdown was performed as described and DNA/RNA was extracted using the DNeasy/RNeasy kits (Qiagen, Crawley, UK), respectively. Total RNA was reverse transcribed using the Quantitect RT kit (Qiagen, Crawley, UK). Pyrosequencing primers were designed using Pyromark Assay Design 2.0 software (Qiagen, Crawley, UK) to measure the DNA methylation levels of *ESR1* and *PGR* after ATF2 knockdown and synthesised by Eurofins MWG Operon (Ebersberg, Germany). The primer sequences are listed in Supplementary Material and Methods, Table 3. Genomic DNA was treated with sodium bisulphite using the EZ DNA methylation Kit (Zymo Research, CA, USA). PCR amplifications were performed in a final volume of 25 μl using HotStarTaq Master Mix (Qiagen, Crawley, UK), 200 nM biotinylated primer, 400 nM non-biotinylated primer and 60 ng of bisulfite-treated genomic DNA. The thermal profile was 95 °C for 5 min followed by 40 cycles of denaturation at 94 °C for 30 s, annealing at 51–56 °C for 30 s and extension at 72 °C for 30 s. The PyroMark Gold Q96 SQA Reagents and the PyroMark Q96 ID instrument (Qiagen, Crawley, UK) were used for pyrosequencing analysis following the supplier’s protocol. The methylation index for each promoter was calculated as the mean value of mC/(mC + C), where C is unmethylated cytosine and mC is 50′ methyl-cytosine, for all examined CpGs in the target sequence.

### Statistical methods

Student’s *t* test (two-sided) was used to compare any differences observed between each experimental group and the control group. All the *t* tests and the 95% CI (confidence interval) were calculated by GraphPad Prism 5. A *p* value less than 0.05 was considered significant.

## Results

### Knockdown of ATF2 by siRNA interferences has differential effect on the growth and proliferation of tamoxifen-sensitive MCF7 and resistant TAMR cell lines

The expression of ATF2 was initially assessed in parental MCF7 cells and its tamoxifen-resistant subline TAMR. There were no significant differences observed at the protein and RNA expression levels between MCF7 and TAMR cells (Supplementary Fig. 1A,B). However, as previously reported, TAMR cells did exhibit increased HER-2 protein levels when compared to MCF7 cells and enhanced ERK1/2 activity (increased pERK1/2, Supplementary Fig. 1A) [28]. In addition, while in MCF7 cells, tamoxifen inhibited E2-induced growth (Supplementary Fig. 1C), TAMR cells grew both in the absence of E2 (vehicle) and in the presence of tamoxifen (Supplementary Fig. 1D) confirming that TAMR cells were both oestrogen-independent and tamoxifen-resistant. Moreover, there was no difference observed in the phosphorylation status of ATF2 in vitro between MCF7 and TAMR cells before or after siATF2 (Supplementary Fig. 1E).

**Fig. 1:**
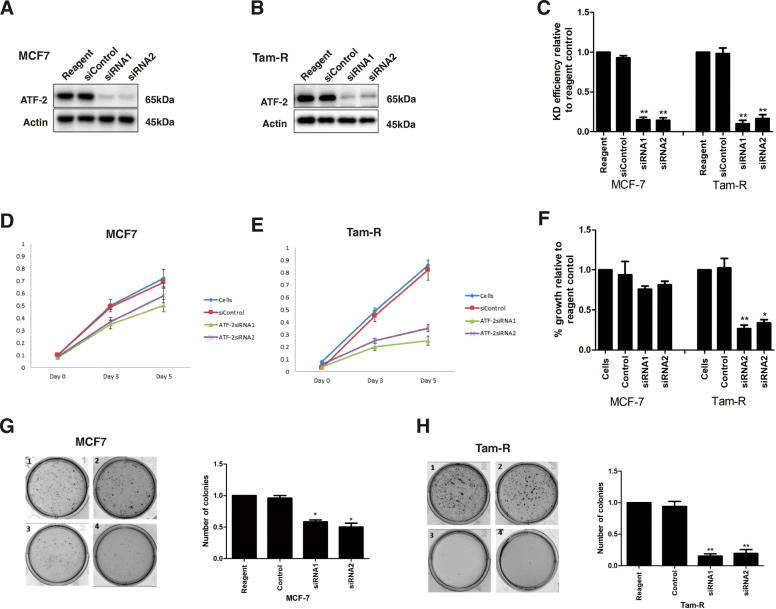
Effect of knockdown of ATF2 on growth and tumourigenesis of MCF7 and TAMR (MCF7-derived tamoxifen-resistant) cell lines. MCF7 (**a**) and TAMR (**b**) cells were transfected with negative control siRNA (siControl), ATF2-siRNA1 and ATF2-siRNA2. Protein lysates were prepared and immunoblotting was carried out for ATF2 with β-actin as a loading control. **c** Densitometry analysis of knockdown efficiency relative to untransfected cells. SRB growth assay for MCF7 (**d**) and TAMR (**e**) indicated a significant growth reduction in TAMR cells after ATF2 silencing (*n* = 3). **f** Graph indicating the percentage of growth at day 5 relative to untransfected cells. **g** The effect of ATF2 knockdown on tumourigenesis was determined by anchorage-independent colony formation. Although there was a reduction of colonies in the MCF7 cells after ATF2 silencing, this reduction was more profound in the TAMRs (*n* = 3). Asterisks indicate statistically significant (*p < 0.05, **p < 0.005) difference from untransfected cells

Loss of ATF2 in both the cell lines by siRNA was confirmed by immunoblotting (Fig. 1a–c) and the growth of cells was determined by the SRB assay (Fig. 1d–f). The efficiency of transfection (densitometry analysis) is presented in Fig. 1c indicating a significant reduction in ATF2 after siRNA in both MCF7 and TAMR cells. While in MCF7 cells there was no significant difference in growth relative to untransfected cells or cells transfected with control siRNA (siControl), knockdown of ATF2 was associated with strong growth inhibition in TAMR cells (Fig. 1f: *p* = 0.0037 ATF2-siRNA1 and *p* = 0.0041 ATF2-siRNA2, day 5). To further validate our observations in the ATF2 dependency of tamoxifen-resistant breast cancer cells, ATF2 knockdown was performed in the independently derived endocrine-resistant MCF7 cells, LCC2 and LCC9 [29, 30] (Supplementary Fig. 2a–f) and showed similar results to the TAMR cells; ATF2 silencing significantly inhibited the growth of both LCC2 and LCC9 cell lines.

Moreover, a hallmark of carcinogenesis is the ability of transformed cells to grow independently of a solid surface, known as anchorage-independent growth. To investigate the effect of ATF2 in anchorage-independent growth of MCF7s and TAMRs, we performed the soft-agar colony formation assay after siRNA-mediated knockdown of ATF2. While ATF2 knockdown inhibited colony formation in MCF7 cells (Fig. 1g), knockdown of ATF2 was associated with a substantially greater reduction in colony formation in TAMR cells (*p* = 0.0016 for siRNA1 and 0.0056 for siRNA2) relative to control (Fig. 1h). To further validate this observation, ATF2 knockdown was performed in LCC2 and LCC9 (Supplementary Fig. 2G). The results were similar to those obtained for TAMR cells. This indicated that although ATF2 was important for anchorage-independent cell growth in both MCF7 and TAMRs, the effect was more profound on tamoxifen-resistant breast cancer cell lines, suggesting that ATF2 targets and regulates additional pathways associated with the tamoxifen resistance phenotype.

### Knockdown of ATF2 by siRNA alters the chemotactic and migratory properties of the resistant TAMR cell lines

**Fig. 2:**
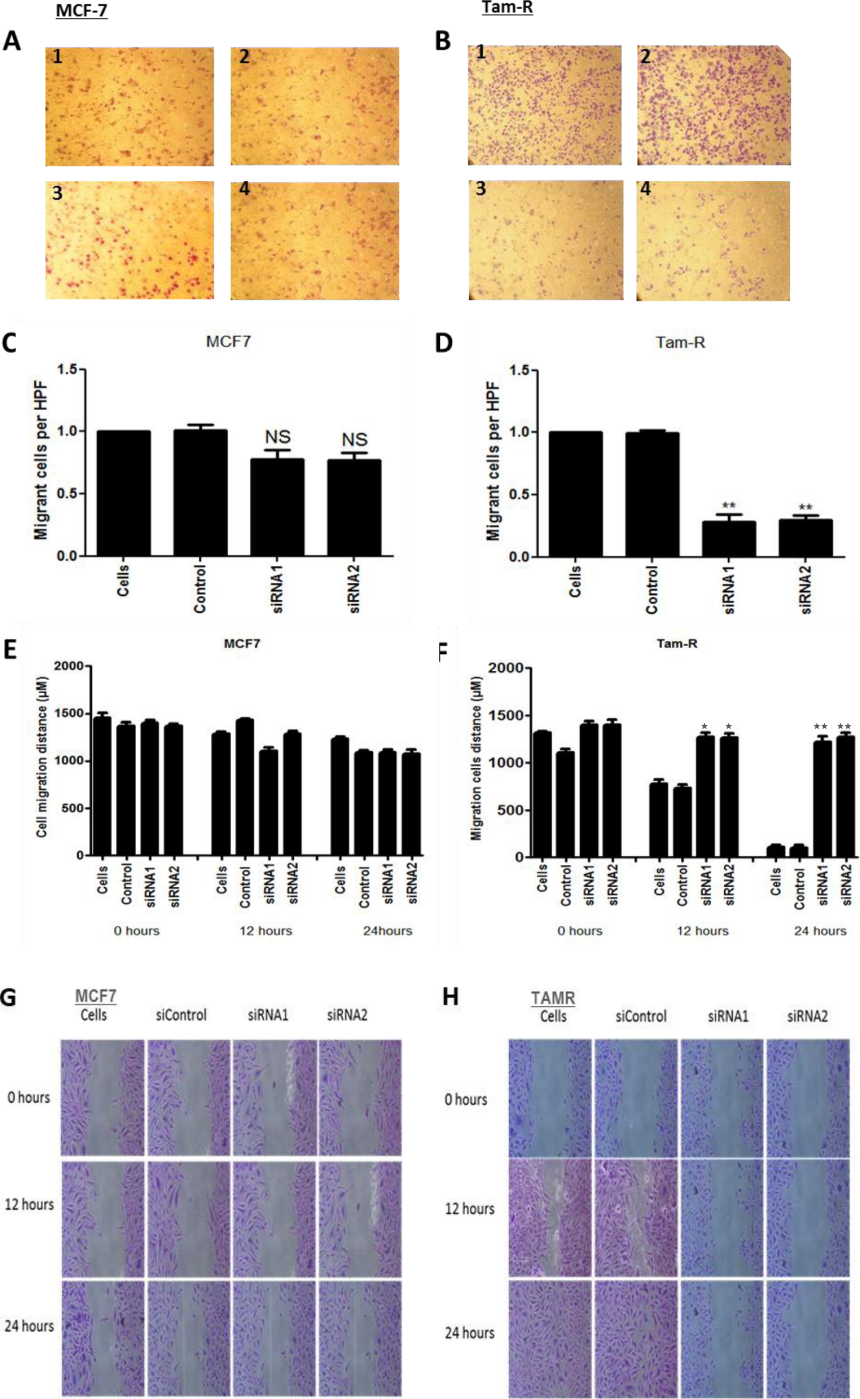
Effect of knockdown of ATF2 on migration of MCF7 and MCF7 derived tamoxifen-resistant cell line (TAMR). MCF7 (**a**, **c**, **e**, and **g**) and TAMR (**b**, **d**, **f**, and **h**) cells transfected with negative control siRNA (siControl), ATF2-siRNA1 and ATF2-siRNA2. Chemotaxis assay (migration) was carried out and migrated cells were counted relative to vehicle control (*n* = 3). ATF2 knockdown inhibited the migratory ability of TAMR (**b**, **d**) cells but not of the MCF7 (**a**, **c**). Similarly, knockdown of ATF2 reduced the migratory ability of TAMR (**f**, **h**) cells in an in vitro wound healing scratch assay but did not affect the MCF7 (**e**, **g**) cells. Asterisks indicate statistically significant (*p < 0.05, **p < 0.005) difference from vehicle control

**Fig. 3:**
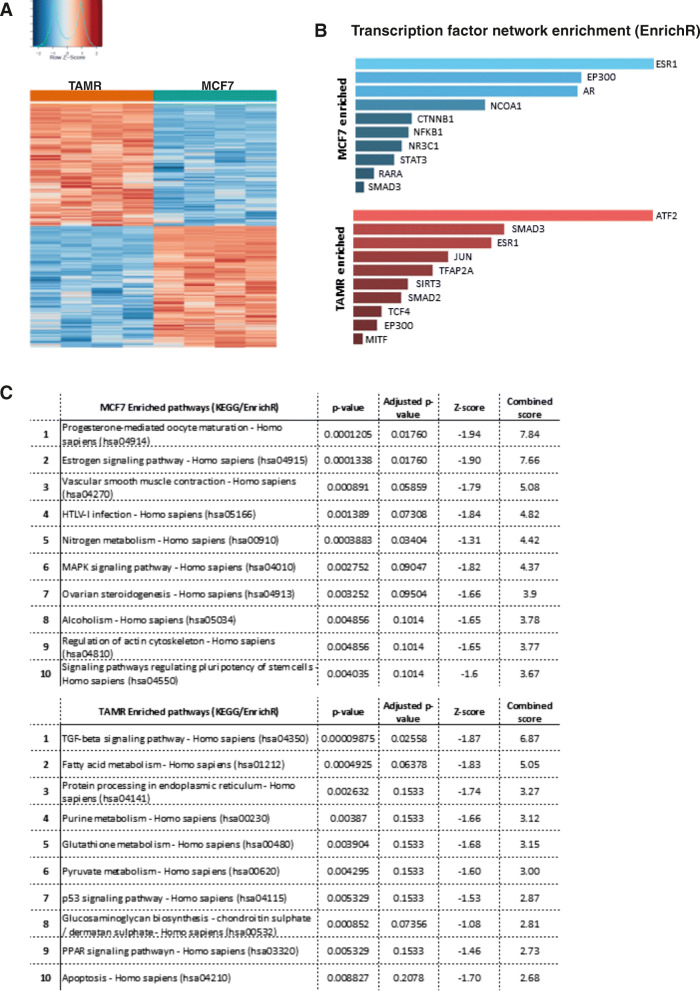
Differential gene expression analysis in basal MCF7 versus TAMR cell lines revealed an ATF2-enriched transcription factor network. **a** Heatmap of genes that were differentially expressed in MCF7 (blue) relative to TAMR (red) cells at basal level. This analysis identified 3260 upregulated and 4423 downregulated genes in TAMR cells relative to MCF7. **b** Enrichment analysis identified a shift from the ER (ESR1)-enriched transcription factor network present on MCF7 (blue) towards an ATF2-enriched transcription factor network on TAMR (red). **c** KEGG enrichment also identified a shift from the oestrogen and progesterone pathways to TGF-β and metabolic pathways

To investigate the role of ATF2 on invasion and migration of breast cancer cell lines, knockdown of ATF2 was performed in both MCF7 and TAMR cells growing in their standard growth media. The invasive and migratory ability of MCF7 and TAMR cells after ATF2 knockdown was determined by chemotaxis (Fig. 2a–d) and wound healing scratch assays (Fig. 2e–h). While in MCF7 cells there was no significant difference in cell migration relative to vehicle control (Fig. 3a, c), knockdown of ATF2 was associated with a significant inhibition of migration in TAMR cells (Fig. 3b, d: *p* = 0.0075 ATF2-siRNA1 and *p* = 0.0025 ATF2-siRNA2). To further validate this observation, wound healing scratch assay was performed. ImageJ software was used to measure the migration ability of both MCF7 (Fig. 2e, g) and TAMR (Fig. 2f, h) cells. Knockdown of ATF2 significantly reduced the migratory capacity of the invasive TAMR cells (*p* = 0.0010 ATF2-siRNA1 and *p* = 0.0015 ATF2-siRNA2 at 12 h and *p* < 0.0001 with both ATF2-siRNA1 and ATF2-siRNA2 at 24 h), whereas no effect was observed in the less invasive MCF7 cells. Similar impairment in cell invasion and migration was obtained in LCC2 and LCC9 cell lines after ATF2 silencing (Supplementary Fig. 3A-F). This indicates that ATF2 plays an important role in regulating migration of tamoxifen-resistant breast cancer cells, compared to endocrine-sensitive MCF7 cells.

### Differential gene expression analysis in MCF7 versus TAMR cell lines revealed a shift from the ER-enriched transcription factor network towards an ATF2-enriched

To identify the underlying mechanisms by which ATF2 regulates the growth and migratory ability of TAMR cells, global gene expression analysis (microarray profiling using Illumina Human HT12 v4 arrays) was carried out both in MCF7 and TAMR before and after ATF2 knockdown including NT-siRNA as control. Differential expression analysis was carried out using limma to identify, firstly, genes that were differentially expressed in MCF7 and TAMR and, secondly, genes that were differentially regulated by ATF2 in TAMR relative to MCF7.

Initial gene expression analysis between MCF7 and TAMR cells identified 3260 genes that were significantly upregulated (FDR < 0.05) and 4423 genes that were significantly downregulated (FDR < 0.05) (Fig. 3a) in MCF7 cells relative to TAMR (full list of genes and pathway enrichment analysis is provided in Supplementary file 1). Functional protein association analysis using the STRING database showed that MCF7 cells are enriched for genes involved in oestrogen and nuclear receptor signalling, cell cycle, lysosomal pathway and endocytosis. The lysosomal degradation pathway regulates a variety of cellular functions such as autophagy, endocytosis and phagocytosis to maintain cellular homeostasis, and lysosomes are also involved in the regulation of ERα signalling pathways that mediate physiological hormone-induced effects [34]. On the other hand, TAMR cells are enriched for genes involved in metabolic pathways (mitochondrial metabolism, ATP production, fatty acid metabolism and RNA processing) as previously observed [10, 11, 35].

Transcription factor network enrichment analysis (EnrichR) indicated an *ESR1* and ER-regulatory gene enrichment in MCF7 cells that were diminished in TAMR (Fig. 3b, c with progesterone and oestrogen signalling adjusted *p* = 0.017). A shift to an ATF2-enriched network and TGF-β signalling pathway was observed in TAMR cells (Fig. 3b, c with TGF-β signalling adjusted *p* = 0.025) suggesting the importance of alternative hormone-independent growth mechanisms.

### Differential gene expression analysis after ATF2 silencing revealed ER-responsive genes and pathways regulated by ATF2 in tamoxifen resistance

**Fig. 4:**
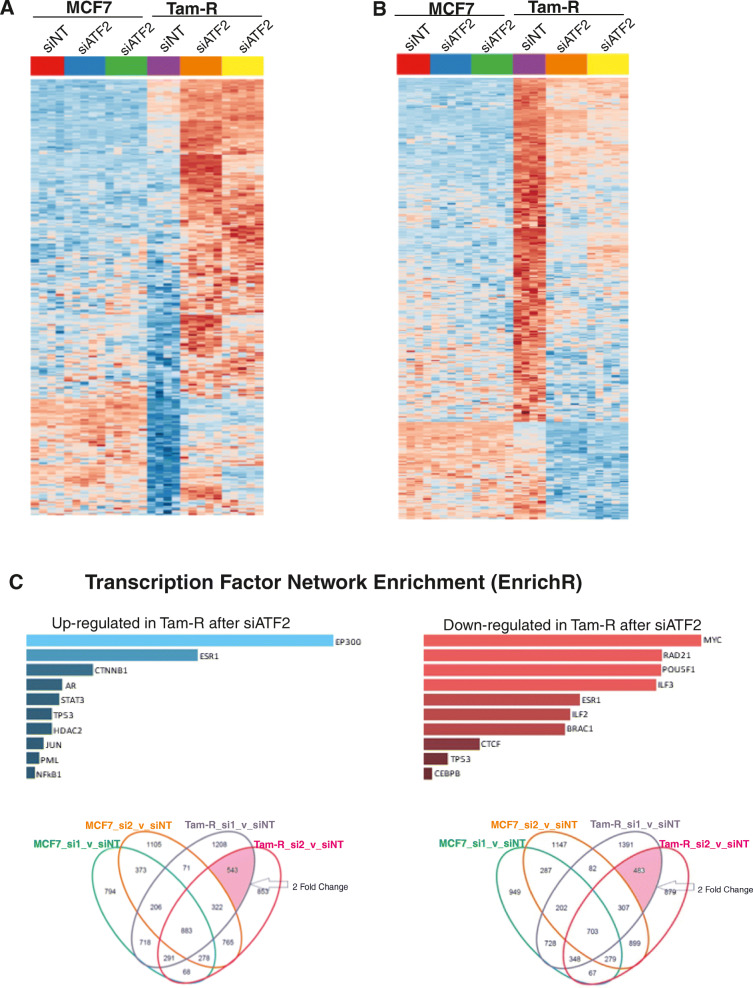
Differential gene expression analysis after ATF2 silencing revealed ER-responsive genes and pathways regulated by ATF2 in TAMR. Heatmaps of genes that were **a** upregulated and **b** downregulated in TAMRs (red) cells after ATF2 silencing relative to MCF7 (blue). **c** Enrichment analysis revealed ATF2 silencing in TAMR cells leads to the reintroduction of the ER signalling network and a reduction in MYC. Venn diagrams of differentially regulated genes in TAMRs after ATF2 silencing. A total of 543 upregulated (**d**) and 483 downregulated (**e**) genes were commonly identified after both ATF2-siRNA knockdown (si1 and si2 vs siNT)

To identify genes regulated by ATF2, we performed differential gene expression analysis in MCF7 and TAMR cell lines after knocking down ATF2 with two independent siRNAs (Fig. 4a, b). Differential analysis in MCF7 cells identified 373 genes up- and 287 downregulated after ATF2 knockdown (Venn diagram Fig. 4c). In contrast, ATF2 knockdown in TAMR cells led to a substantially greater number of genes differentially expressed with 543 upregulated and 483 downregulated genes (Venn diagram Fig. 4c) (full list of genes and pathway enrichment analysis is provided in Supplementary file 2). Focusing on differentially expressed genes specific to the TAMR cell line (without changing in MCF7), we found a reintroduction of ER and its signalling pathway upon ATF2 knockdown along with a reduction in MYC transcriptional activity by EnrichR (Fig. 4c). Similarly, functional protein association analysis using the STRING database on TAMR-upregulated genes after ATF2 knockdown showed an enrichment for adherent junctions, drug metabolising enzymes, steroid hormone biosynthesis, PTEN regulation, oestrogen and nuclear receptor signalling. In addition, pathway enrichment analysis on TAMR-downregulated genes after ATF2 knockdown showed enrichment for ribosomal pathway (rRNA processing in the nucleolus and cytosol and RNA metabolism). Table 1 summarises the pathways that are differentially regulated in MCF7 versus TAMR and in TAMR after ATF2 silencing. Since we observed that MCF7 cells are enriched for genes involved in oestrogen and nuclear receptor signalling in comparison to the TAMRs and that these two pathways were upregulated in TAMRs after ATF2 silencing, we focused to investigate further the effect of ATF2 silencing in ER-regulated genes.

**Table 1:** Pathways that are differentially regulated in MCF7 versus TAMR and in TAMR after ATF2 silencing. Commonly identified pathways dysregulated between MCF7 and TAMRs and re-instated after ATF2 knockdown. The complete list of KEGG and REACTOME pathways are presented in Supplementary files 1 (MCF7 vs TAMR) and 2 (TAMRs after siATF2 knockout)

Number term ID	Term description
**Pathways upregulated in MCF7 (downregulated in TAMRs)**	
HSA-9018519	Oestrogen-dependent gene expression
HSA-9006931	Signalling by Nuclear Receptors
**Pathways upregulated in TAMRs after siATF2**	
HSA-9018519	Oestrogen-dependent gene expression
HSA-9006931	Signalling by Nuclear Receptors
**Pathways downregulated in MCF7 (upregulated in TAMRs)**	
hsa03010	Ribosome
HSA-8953854	Metabolism of RNA
HSA-6791226	Major pathway of rRNA processing in the nucleolus and cytosol
HSA-1799339	SRP-dependent cotranslational protein targeting to membrane
HSA-71291	Metabolism of amino acids and derivatives
**Pathways downregulated in TAMRs after siATF2**	
hsa03010	Ribosome
HSA-8953854	Metabolism of RNA
HSA-6791226	Major pathway of rRNA processing in the nucleolus and cytosol
HSA-1799339	SRP-dependent cotranslational protein targeting to membrane
HSA-71291	Metabolism of amino acids and derivatives

**Fig. 5:**
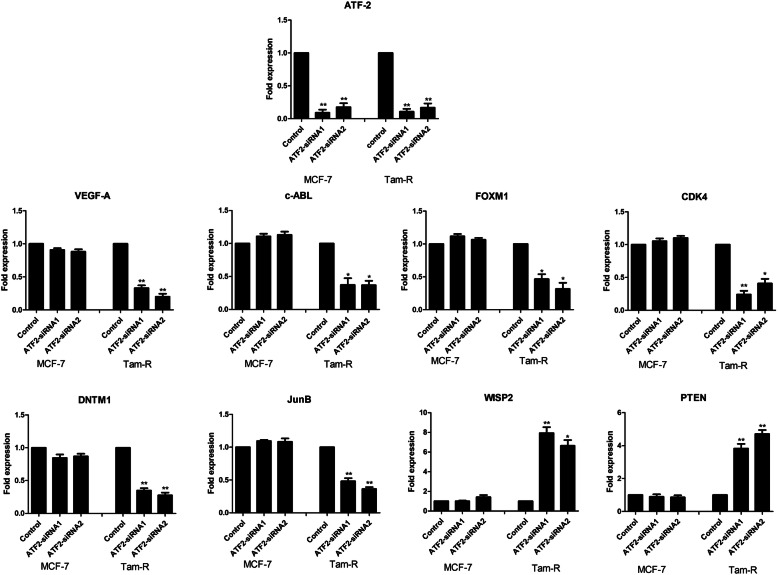
Validation of genes identified using microarray. MCF7 and TAMR cells were transfected with silencer negative control siRNA, ATF2 siRNA1 and ATF2 siRNA2. qRT-PCR was carried out using Taqman primers for the genes indicated. Data analysis for each cell line was carried out relative to the siControl (*n* = 3) and asterisks indicate the genes that were significantly changed after ATF2 knockdown (*p < 0.05, **p < 0.005)

**Fig. 6:**
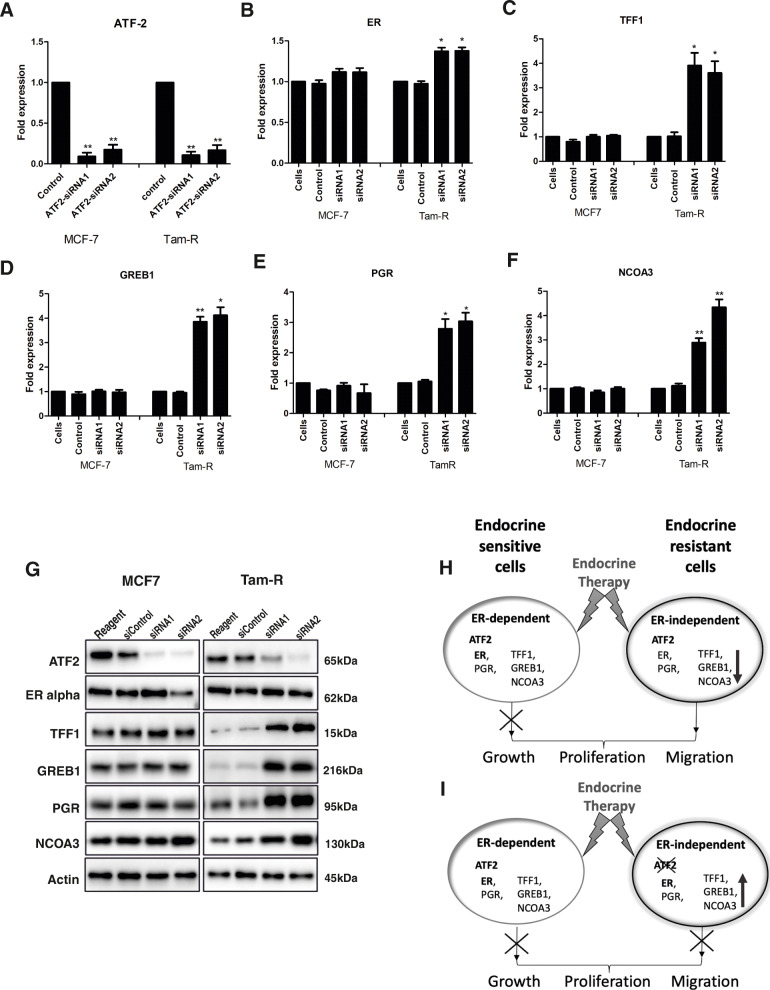
Effect of knockdown of ATF2 on ER and ER-regulated genes and proposed model of resistance. **a**–**f** MCF7 (left bars) and TAMR (right bars) cells were transfected with ATF2 siRNA and qRT-PCR was carried for *ATF2* (**a**), ERα (*ESR1*) (**b**), *TFF1* (**c**), *GREB1* (**d**), *PGR* (**e**) and *NCOA3* (**f**) using TaqMan primers. *GAPDH* was used as the housekeeping gene and changes in mRNA levels after ATF2 knockdown were calculated relative to the control. Asterisks indicate the genes that were significantly changed after ATF2 knockdown (*p < 0.05, **p < 0.005). **g** Protein lysates were also prepared in triplicates of three independent experiments and immunoblotting was carried out using the antibodies indicated. ATF2 knockdown had no effect on the ER and ER-regulated genes on the tamoxifen-sensitive MCF7 cells but affected their expression both on the mRNA and protein levels on the tamoxifen-resistant TAMRs. The following model of resistance is proposed: **h** In endocrine-sensitive cells, gene transcription is ER-dependent and endocrine therapy is able to stop their growth and proliferation. However, endocrine-resistant cells have a shift from ER-dependent to an ER-independent ATF2-dependent transcriptional program and therefore, they are not responding to endocrine treatment. **i** Targeting the ATF-2 transcription factor in endocrine-resistant cells represents a new mechanism to revert resistance and enhance endocrine sensitivity. The genes shown in this model (**h**, **i**) such as TFF1, GREB1 and NCOA3 are examples of resistance candidates only, since their biological function to endocrine resistance has not been validated in animal models in this study

Firstly, to confirm the gene lists obtained from the microarray data analysis, qRT-PCR was carried out using specified TaqMan primers. A number of the ER-regulatory key genes that were significantly up- or downregulated in TAMR after ATF2 knockdown presented similar results by qRT-PCR (Fig. 5). To further evaluate the effect of ATF2 knockdown on ER expression and activity at both RNA and protein level, qRT-PCR and western blotting were performed for the *ESR1*(ER*)* and ER-regulated genes *TFF1*, *PGR*, *GREB1* and *NCOA3* (Fig. 6a–g). With the exception of *PGR*, TAMR cells had a reduced expression of the ER-regulated genes *TFF1*, *GREB1* and *NCOA3* compared to tamoxifen-sensitive MCF7. Knockdown of ATF2 resulted in increased expression of *ER* and its targets *TFF1*, *GREB1*, *NCOA3* and *PGR* in TAMR cells but not in MCF7 cells in both the gene and protein levels. This indicated that knockdown of ATF2 in TAMR cells enhances ER expression and activity which confirmed our earlier results.

Moreover, the list of genes differentially expressed in TAMRs after siATF2 was assessed by the Drug-Gene interactions database (DGIdb) v3.1 (http://www.dgidb.org/) and presented in supplementary file 3. Silencing of ATF2 in TAMRs lead to downregulation of ABL1, CDK4, CTGF, DNMT1, PARP3 and VEGFA that have been linked to endocrine resistance and poor prognosis in BC, and there are several drugs available targeting these genes (FDA approved or in clinical trials, supplementary file 3). In addition, silencing of ATF2 lead to upregulation of genes such as PTEN and IGF1R.

### DNA methylation analysis

DNA methylation levels of *ESR1* and *PGR* were assessed following ATF2 knockdown in parental MCF7 and tamoxifen resistance TAMR, LCC2 and LCC9. The threshold for scoring hypermethylated samples was conservatively set to 10% [36]. *ESR1* and *PGR* promoters were unmethylated in both parental and ATF2 knockdown derivatives. In addition, global methylation in MCF7, TAMR, LCC2 and LCC9 in presence or absence of ATF2 was measured by assessing long interspersed nucleotide element (LINE-1) methylation [37]. Global methylation levels are shown in detail in Supplementary Fig. 4A. Tamoxifen resistant cells showed slightly increased global methylation compared to parental MCF7 at the basal level. However, ATF2 knockdown did not result in any significant changes (*p* > 0.05 in all comparisons) in any of the cell lines tested. Representative pyrograms of MCF7 and TAMRs are given in supplementary Fig. 4B,C.

## Discussion

The development of endocrine therapy resistance appears to involve multiple divergent mechanisms including epigenetic changes affecting the expression of ER and its target genes or deregulated ER-mediated gene transcription [3–10]. Therefore, a better understanding of the complexity of resistance will help us to identify novel targets able to revert or abolish resistance to endocrine therapy. Studies utilising cell lines and patient-derived xenografts models of acquired resistance have indicated impaired ER-mediated transcriptional activity, enrichment for genes involved in cell growth, cell survival and apoptosis and, more recently, activation of oestrogen non-genomic signalling, involving mainly the ERa/Src/PI3K complex [38–41]. Blocking the formation of the ERa/Src/PI3K complex by a competitive peptide partially restored tamoxifen sensitivity in the resistant cells [41]. Moreover, blocking the AP1 transcription complex (Jun, Fos, Maf and ATF2 protein subfamilies) leads to increased tumour sensitivity to endocrine therapy and delayed onset of resistance through the inhibition of both proliferative and survival signals [40]. A recent study highlighted that ATF2 promotes the development and progression of urothelial cancer via cooperation with androgen receptor and EGFR/ERBB2/ERK pathway signalling and suggested that ATF2 inhibition in association with AR inactivation may be a potential effective therapeutic approach for urothelial cancer [42]. Although there is conflicting data regarding the prognostic role of AR expression in endocrine treatment response, an in vitro effect of AR expression on tamoxifen resistance has been observed, potentially mediated through activation of EGFR signalling pathways and the adaptation of the tumour cells from oestrogen to androgen dependent has been demonstrated as another mechanism of resistance to ER targeted therapies [13, 43, 44]. Therefore, silencing ATF2 may be beneficial to endocrine therapy resistance.

In a previous study from our group, we showed that ATF2 silencing leads to a loss in the growth-inhibitory effects of tamoxifen in the ER-positive, tamoxifen-sensitive MCF7 cell line and that tamoxifen treatment caused a dose-dependent phosphorylation of ATF2 within its activation domain, enhancing its transcriptional activity, suggesting a tumour-suppressive role of ATF2 in ER-positive breast cancer [25]. In the current study, we demonstrated in vitro that the transcription factor ATF2, part of the AP1 complex, represents a key factor in acquired endocrine resistance by facilitating a shift towards ER-independent transcription and silencing of ATF2 leads to the reversal of this resistant phenotype.

Firstly, we demonstrated that knockdown of ATF2 had a differential effect on the growth and proliferation of MCF7 and the endocrine-resistant cell lines TAMR, LCC2 and LCC9. Interestingly, transient knockdown of ATF2 significantly inhibited the growth of TAMR, LCC2 and LCC9 but had little effect on the growth of MCF7 cells. In addition, ATF2 silencing was also associated with a significant reduction in colony formation in the endocrine-resistant cell lines in comparison to the MCF7-sensitive cells. These results indicate that ATF2 plays an important role in regulating growth of tamoxifen-resistant breast cancer cell lines, without affecting the growth of the tamoxifen-sensitive cells, by altering signalling pathways that lead to reduced cell proliferation and/or increased apoptosis and by affecting their anchorage-independent growth and tumorigenic potential. The effect of siATF2 in the induction of apoptosis in MCF7 and TAMR cells is presented in supplementary Fig. 5 whereas the lack of difference in the phosphorylation status of ATF2 in vitro between MCF7 and TAMR cells before or after siATF2 (supplementary Fig. 1E) highlights further the effect of ATF2 as a transcription factor and not due to its activity. We previously showed that high expression of p-ATF2 (69/71) was associated with longer disease-free (DSS) and breast cancer-specific survival (BCSS) in ER-positive high-risk patients exposed to tamoxifen [25]. However, these patients do not represent a tamoxifen-resistant model to compare it to the current in vitro work. Our data is similar to a number of studies mainly in melanoma, pancreatic and oesophageal cancer where inhibition or silencing of ATF2 leads to an induction of apoptosis and inhibition of tumour growth and metastasis [45–48].

We further showed that silencing ATF2 alters the chemotactic and migratory properties of the resistant cell lines and reverts the migratory capacity of the invasive TAMR, LCC2 and LCC9 cells with no effect in the less invasive MCF7 cells. This highlights further the distinct transcriptional activities between the endocrine resistant and sensitive cells and the importance of ATF2 in reverting the malignant phenotype and restoring sensitivity to endocrine therapy.

The mechanisms by which ATF2 regulates the growth and migratory ability of TAMR cells in comparison with MCF7 was evaluated by microarray gene expression analysis of both cell lines before and after ATF2 silencing. Following ATF2 silencing, analysis was carried out to identify genes that were differentially expressed in TAMR cells without changing in MCF7. This revealed a number of ER-responsive genes and pathways regulated by ATF2 in tamoxifen resistance and highlighted a shift from the ER-enriched towards an ATF2-enriched transcription factor network. Although the endogenous levels of ATF2 were not found to be significantly different between the cell lines according to our microarray data (reconfirmed by qRT-PCR and western blot), enhanced expression of *ATF3* and *ATF4* (members of the ATF family) was observed in TAMRs, suggesting enhanced expression and activity of ATF transcription factors in tamoxifen resistance. Knockdown of ATF2 in TAMR cells differentially inhibited the expression of genes that are well documented in the literature to play key roles in mediating tamoxifen resistance such as *VEGFA*, *ABL1*, *FOXM1* and *DNMT1* [3, 7, 11, 12, 49, 50]. Moreover, ATF2 knockdown also inhibited the expression of *CDK4* and *ATF3* which were found to be overexpressed in TAMR cells relative to MCF7. Many of the genes differentially expressed in TAMRs cells after ATF2 silencing (Supplementary file 3) are drug targets and could be potentially used to overcome the endocrine-resistant phenotype. For instance, inhibition of ABL in ER-positive breast cancer resulted in sensitization to anti-oestrogen therapies [49, 51, 52] whereas CDK4/6 inhibitors have demonstrated clear clinical efficacy in the treatment of ER-positive, HER2-negative breast cancer when combined with endocrine therapy [53, 54]. DNMT1 overexpression in TAMR cells leads to aberrant methylation of the PTEN promoter resulting into loss of PTEN expression and activation of the PI3K/AKT pathway. Therefore, drugs targeting DNMT1 (azacitidine, decitabine) may be of potential clinical use [55, 56]. CTGF overexpression has been correlated to decreased survival and endocrine resistance in ER-positive breast cancer patients and identified as a potential therapeutic target to overcome resistance [57] whereas ruxolitinib blocked the EMT process and VEGF production through the JAK/STAT3 pathway, consequently suppressing tamoxifen-resistant cell migration and angiogenesis [58]. We also demonstrated IGF1R expression was upregulated after silencing ATF2. Of note, a randomised study targeting IGF1R with ganitumab in combination with endocrine therapy for hormone-receptor positive locally advanced or metastatic breast cancer demonstrated a significantly worse overall survival in those women receiving ganitumab as compared to placebo [59]. Previously, it had been reported that IGF1R is reduced in tumour biopsy at the time of recurrence or resistance to tamoxifen and time to progression was significantly increased for IGF1R rich patients [60]. These observations alongside ours would support a role for agonising IGF1R in ER-positive breast cancer.

Knockdown of ATF2 also resulted in increased expression of *ESR1* and ER-target genes, including *TFF1*, *GREB1*, *NCOA3* and *PGR* in TAMR cells but not in MCF7 cells. This was also reconfirmed in the protein levels indicating that knockdown of ATF2 in TAMR cells enhances ER-regulated gene expression and activity. Using the Kaplan-Meier Plotter (https://kmplot.com/analysis/) [61] for breast cancer we observed that mRNA expression of NCOA3, GREB1 but not ATF2 and TFF1 correlated with progression-free survival (PFS) whereas NCOA3, GREB1 and TFF1 correlated with overall survival (OS) in ER-positive breast cancer patients systemically-treated with tamoxifen only (Supplementary Fig. 6). The KM plotter data for ATF2 is in agreement with our previous work utilising clinical samples [25]. However, it cannot distinguish between tamoxifen-sensitive and tamoxifen-resistant patients. In the current study, we did not examine any clinical samples as out work was purely in vitro models of endocrine resistance.

Pathway analysis of genes upregulated in TAMRs after ATF2 silencing showed very strong enrichment for adherens junction, oestrogen and nuclear receptor signalling and drug metabolism whereas similar analysis of genes downregulated in TAMRs after ATF2 silencing showed enrichment for the ribosome (Supplementary files 1 and 2). The pathway analysis confirms our functional studies of anchorage-independent cell growth and migration and is in agreement with a previous study that observed an increased activity of mitochondrial biogenesis and expression of ribosomal genes in the anchorage-independent cell growth gene signature [62].

Finally, DNA methylation has been implicated in the development of tamoxifen resistance [6, 7]. However, in our study, although tamoxifen-resistant cells showed slightly increased global methylation compared to parental MCF7 at basal level, ATF2 knockdown did not trigger any significant changes indicating that ATF2 does not affect the methylation profile of these cell lines.

## Conclusion

Our work confirms previous studies showing that tumour cells adapt to oestrogen deprivation during treatment with tamoxifen and/or AIs by the activation of alternate signalling pathways and that abrogation of alternative growth factor signalling may restore sensitivity to endocrine therapy. Moreover, as we previously showed that ATF2 plays a tumour-suppressive role in ER-positive endocrine sensitive breast cancer, the current data on an in vitro endocrine-resistant model highlights the dual role of ATF2 in breast cancer and suggests that ATF2 targeting has the potential of being effective novel therapeutic approach in endocrine-resistant breast cancer. Therefore, based on the in vitro data and pathway analysis presented in this study, we propose the following model presented in Fig. 6h, i: In endocrine-resistant cells, where the classical ER-regulated model of gene transcription is not the dominant route, targeting the ATF-2 transcription factor or its key downstream signalling targets represents a new mechanism to revert resistance and enhance sensitivity to endocrine therapy. However, additional genes and pathways affected by ATF2 may interfere and/or contribute to this model and further in vivo work could enhance our knowledge into the mechanistic action of ATF2.

## Supplementary Information

**Additional file 1: Supplementary Table 1.** Taqman assays used in the study. **Supplementary Table 2.** Details of the antibodies used in the study. **Supplementary Table 3.** The primer sequences used for DNA methylation analysis.

**Additional file 2: Supplementary Fig. 1.** Validation of ATF2 expression in MCF7 and TAMR cells and the effect of ATF2 knockdown. The protein **(A)** and mRNA **(B)** expression of ATF2 was similar between MCF7 and TAMR cell lines. However, **(A)** TAMRs also showed increased protein expression of HER2 and enhanced ERK1/2 activity. SRB growth assay was performed on MCF7 **(C)** and TAMR **(D)** cells treated with vehicle, estradiol (E2) (10 nM), 4-hydroxytamoxifen (Tam) (100 nM) or both E2 and Tam. Tamoxifen inhibited E2-induced growth of MCF7 cells whereas, TAMR cells grew both in the absence of E2 and in the presence of tamoxifen confirming their estrogen-independent and tamoxifen-resistant phenotype. **Supplementary Fig. 2.** Effect of ATF2 knockdown on growth and tumourigenesis of MCF7 derived tamoxifen-resistant cell lines LCC2 and LCC9. LCC2 **(A)** and LCC9 **(B)** cells were transfected with negative control siRNA (siControl), ATF2-siRNA1 and ATF2-siRNA2. Protein lysates were prepared and immunoblotting was carried out for ATF2 with β-actin as a loading control. SRB growth assay for LCC2 **(C)** and LCC9 **(D)** indicated a significant growth reduction in both cell lines similar to TAMR cells after ATF2 silencing (*n* = 3). **(E)** Graph indicating the % of growth at day 5 relative to untransfected cells. **(F)** Densitometry analysis of knockdown efficiency relative to untransfected cells. **(G,H)** The effect of ATF2 knockdown on tumourigenesis was determined by anchorage-independent colony formation. There was a reduction of colonies in both the LCC2 (G) and LCC9 (H) cells similar to the TAMRs (n = 3). Asterisks indicate statistically significant difference from untransfected cells (*p < 0.05, **p < 0.005). **Supplementary Fig. 3.** Effect of ATF2 knockdown on migration of MCF7 derived tamoxifen-resistant cell lines LCC2 and LCC9. LCC2 **(A,C,E)** and LCC9 **(B,D,F)** cells were transfected with negative control siRNA (siControl), ATF2-siRNA1 and ATF2-siRNA2. Chemotaxis assay (migration) was carried out and migrated cells were counted relative to vehicle control (n = 3). ATF2 knockdown inhibited the migratory ability of both the LCC2 **(A,C)** and LCC9 **(B,D)** cells. Similarly, knockdown of ATF2 reduced their migratory ability (**E**: LCC2 and **F**: LCC9) in an in vitro wound healing scratch assay with the maximum effect observed at 24 hours. All the experiments were performed in triplicates. Asterisks indicate statistically significant difference from vehicle control (*p < 0.05, **p < 0.005). **Supplementary Fig. 4.** DNA Methylation Analysis. **(A)** Long interspersed nucleotide element (LINE-1) methylation status. Data are presented as percent. **(B)** Representative pyrograms from the DNA methylation analysis of *ESR1* in MCF7 in presence or absence of ATF2 are shown. X axis shows the dispensation order; the examined sequence is shown at the top of each pyrogram. Gray lanes are indicative of individual CG dinucleotides; yellow lanes indicate the bisulfite conversion controls. **(C)** Representative pyrograms from the LINE-1 methylation of MCF7 in presence or absence of ATF2 are shown. X axis shows the dispensation order; the examined sequence is shown at the top of each pyrogram. Gray lanes are indicative of individual CG dinucleotides; yellow lanes indicate the bisulfite conversion controls. **Supplementary Fig. 5.** Effect of ATF2 knockdown on apoptosis of MCF7 and TAMR cells. MCF7 and TAMR cells untreated and treated with siControl and siATF2 were stained with the FITC labelled-Annexin V and propidium iodide (PI) Staining Solution and analyse by FACS. Live cells (L) are FITC-Annexin V−/PI-, early apoptotic cells (A) are FITC-Annexin V+/PI-, whereas late apoptotic cells/necrotic cells (N) are FITC-Annexin V+/PI+ and dead cells (D) are FITC-Annexin V−/PI+. We observed that there was no induction of apoptosis in MCF7 cells (no difference between untreated, siControl and siATF2) but in TAMR cells there was a shift in A (early apoptosis) and N (late apoptosis/necrosis) from 7.70% and 1.52% respectively in the untreated cells to 11.57 and 3.94% after siControl and 17.49% and 18.81% after siATF2 (ATF2 silencing). **Supplementary Fig. 6.** Kaplan-Meier Plot analysis for expression of TFF1, GREB1 or NCOA3, to determine correlation with progression free or overall survival in ER+ patients. Using the Kaplan-Meier Plotter (https://kmplot.com/analysis/) for ER-positive breast cancer patients systemically-treated with tamoxifen only we observed that **(1)** for progression free survival (PFS), mRNA expression of NCOA3 **(B)**, GREB1 **(D)** but not ATF2/CREB2 **(A)** and TFF1/BCEI **(C)** correlated with PFS and **(2)** for overall survival (OS), mRNA expression of NCOA3 **(B)**, TFF1/BCEI **(C)** and GREB1 **(D)** but not ATF2/CREB2 **(A)** correlated with OS.

**Additional file 3: Supplementary file 1.** MvsT_DE Genes and Pathway analysis.

**Additional file 4: Supplementary file 2.** siATF2 MvsT_DE Genes and Pathway analysis.

**Additional file 5: Supplementary file 3.** Drug targets after siATF2 in TAMR.

## Data Availability

All the data generated and analysed in this study is included in the article and its supplementary files. The raw and processed microarray data is deposited at the gene expression omnibus (GEO) database; accession number GSE145548.

## Abbreviations

ATF2: Activating transcription factor-2
ER: Oestrogen receptor
AI: Aromatase inhibitors
CRE: cAMP response element
qRT-PCR: Quantitative real-time polymerase chain reaction
AP-1: Activator protein 1
MMP: Matrix metalloproteinase
DMEM: Dulbecco’s modified Eagle’s medium
FBS: Foetal bovine serum
CSS: Charcoal/dextran-stripped FBS
4-OH-Tam: 4-Hydroxytamoxifen
siRNA: Small interfering RNA
SRB: SulfoRhodamine B
MTT: 3-(4,5-Dimethylthiazol-2-yl)-2,5-Diphenyltetrazolium Bromide
GEO: *Gene Expression Omnibus*
FDR: False discovery rate
LINE-1: Long interspersed nucleotide element-1
