# Supplementary material for: Activating transcription factor-2 (ATF2) is a key determinant of resistance to endocrine treatment in an in vitro model of breast cancer
Source: Breast Cancer Res. 2020 Nov 16;22:126. doi: 10.1186/s13058-020-01359-7 (PMC7667764; doi:10.1186/s13058-020-01359-7)
Supplement: Supplementary file 1 — Additional file 1: Supplementary Table 1. Taqman assays used in the study. Supplementary Table 2. Details of the antibodies used in the study. Supplementary Table 3. The primer sequences used for DNA methylation analysis. [file 13058_2020_1359_MOESM1_ESM.docx]

**Supplementary Material and Methods**

**Supplementary Table 1: Taqman assays used in the study**

| **Gene** | **Primer Code** |
| --- | --- |
| ATF-2 | Hs00153179_m1 |
| c-ABL | Hs01104725_m1 |
| CDK4 | Hs00175935_m1 |
| DNMT1 | Hs02558036_s1 |
| ERα | Hs00174860_m1 |
| FOXM1 | Hs01073586_m1 |
| GREB1 | Hs00536409_m1 |
| JUNB | Hs00357891_s1 |
| PTEN | Hs02621230_s1 |
| TFF1 (pS2) | Hs00170216_m1 |
| VEGF-A | Hs00900055_m1 |
| WISP-2 | Hs01031984_m1 |
| NCOA3 | Hs00180722_m1 |
| PGR | Hs01556702_m1 |
| GAPDH | Hs99999905_m1 |

**Supplementary Table 2: Details of the antibodies used in the study**

| **Antibody** | **Molecular Weight (kDa)** | **Dilution** | **Source** | **Company** |
| --- | --- | --- | --- | --- |
| ATF-2 | 65 | 1:1000 | Rabbit | Cell Signalling |
| p-ATF2 Thr69/71 | 65 | 1:1000 | Rabbit | Cell Signalling |
| ER | 62 | 1:250 | Rabbit | Cell Signalling |
| ERK1/2 | 42&44 | 1:1000 | Rabbit | Cell Signalling |
| GREB1 | 250 | 1:1000 | Rabbit | Cell Signalling |
| HER-2 | 185 | 1:1000 | Rabbit | Cell Signalling |
| pERK1/2 | 42&44 | 1:1000 | Rabbit | Cell Signalling |
| PGR | 118 | 1:1000 | Rabbit | Cell Signalling |
| NCOA3/AIB1 | 155 | 1:1000 | Rabbit | Abcam |
| Β-Actin | 42 | 1:20000 | Mouse | Insight Biotechnology |

**Supplementary Table 3**: **The primer sequences used for DNA methylation analysis**

| **No.** | **Oligo Name** | **Sequence (5'->3'):** |
| --- | --- | --- |
| 1 | PGRmeth-Fb | GAGTTTGATGTTAGAGAAAAAGT (23) |
| 2 | ESRmeth1-Rb | TCCTAAAACTACACTTACTCCC (22) |
| 3 | ESRmeth2-Fb | TTTTTGGGTTATTTTTAGTAGAT (23) |
| 4 | PGRmeth-R | CCAAAAAAATTCTCCAACT (19) |
| 5 | PGRmeth-S | AATTCTCCAACTTCTATCC (19 |
| 6 | ESRmeth1-F | GGGTTATTTGGAAAAAGAGTA (21) |
| 7 | ESRmeth1-S | GTTATTTGGAAAAAGAGTAT (20) |
| 8 | ESRmeth2-R | CCTTCCCTAAACTTTACTTTACTTAT (26) |
| 9 | ESRmeth2-S: | TAAACTTTACTTTACTTATC (20) |
