# Supplementary material for: Activating transcription factor-2 (ATF2) is a key determinant of resistance to endocrine treatment in an in vitro model of breast cancer
Source: Breast Cancer Res. 2020 Nov 16;22:126. doi: 10.1186/s13058-020-01359-7 (PMC7667764; doi:10.1186/s13058-020-01359-7)
Supplement: Supplementary file 2 — Additional file 2: Supplementary Fig. 1. Validation of ATF2 expression in MCF7 and TAMR cells and the effect of ATF2 knockdown. The protein (A) and mRNA (B) expression of ATF2 was similar between MCF7 and TAMR cell lines. However, (A) TAMRs also showed increased protein expression of HER2 and enhanced ERK1/2 activity. SRB growth assay was performed on MCF7 (C) and TAMR (D) cells treated with vehicle, estradiol (E2) (10 nM), 4-hydroxytamoxifen (Tam) (100 nM) or both E2 and Tam. Tamoxifen inhibited E2-induced growth of MCF7 cells whereas, TAMR cells grew both in the absence of E2 and in the presence of tamoxifen confirming their estrogen-independent and tamoxifen-resistant phenotype. Supplementary Fig. 2. Effect of ATF2 knockdown on growth and tumourigenesis of MCF7 derived tamoxifen-resistant cell lines LCC2 and LCC9. LCC2 (A) and LCC9 (B) cells were transfected with negative control siRNA (siControl), ATF2-siRNA1 and ATF2-siRNA2. Protein lysates were prepared and immunoblotting was carried out for ATF2 with β-actin as a loading control. SRB growth assay for LCC2 (C) and LCC9 (D) indicated a significant growth reduction in both cell lines similar to TAMR cells after ATF2 silencing (n = 3). (E) Graph indicating the % of growth at day 5 relative to untransfected cells. (F) Densitometry analysis of knockdown efficiency relative to untransfected cells. (G,H) The effect of ATF2 knockdown on tumourigenesis was determined by anchorage-independent colony formation. There was a reduction of colonies in both the LCC2 (G) and LCC9 (H) cells similar to the TAMRs (n = 3). Asterisks indicate statistically significant difference from untransfected cells (*p < 0.05, **p < 0.005). Supplementary Fig. 3. Effect of ATF2 knockdown on migration of MCF7 derived tamoxifen-resistant cell lines LCC2 and LCC9. LCC2 (A,C,E) and LCC9 (B,D,F) cells were transfected with negative control siRNA (siControl), ATF2-siRNA1 and ATF2-siRNA2. Chemotaxis assay (migration) was carried out and migrated cells [file 13058_2020_1359_MOESM2_ESM.docx]

**Supplementary Figure 1**

**D**

**C**


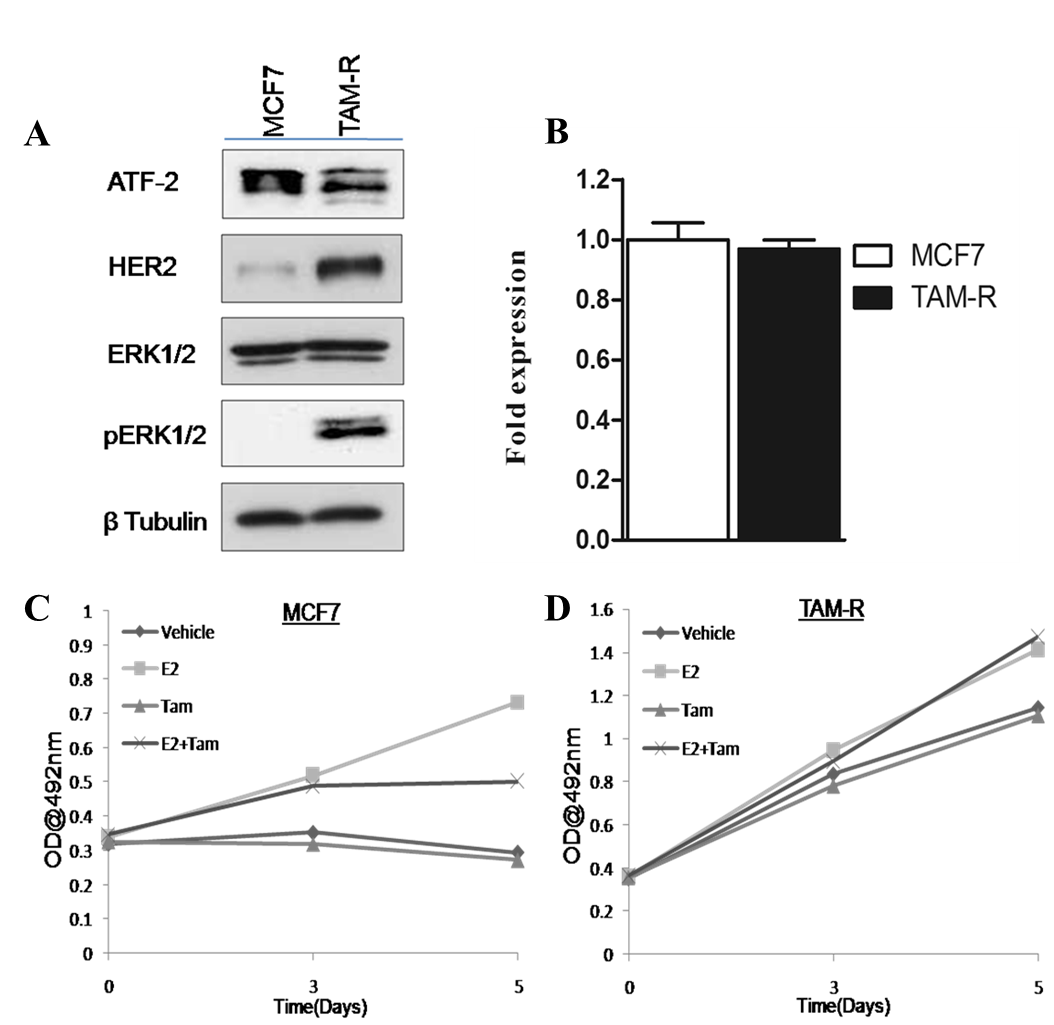


**A**

**B**


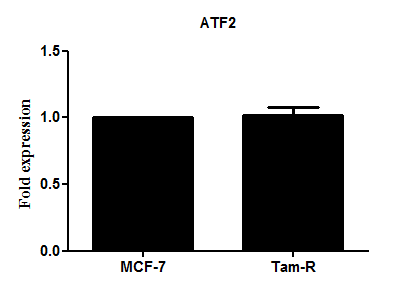


MCF7

TamR

ATF2

HER2

ERK1/2

pERK1/2

β Tubulin

**E**


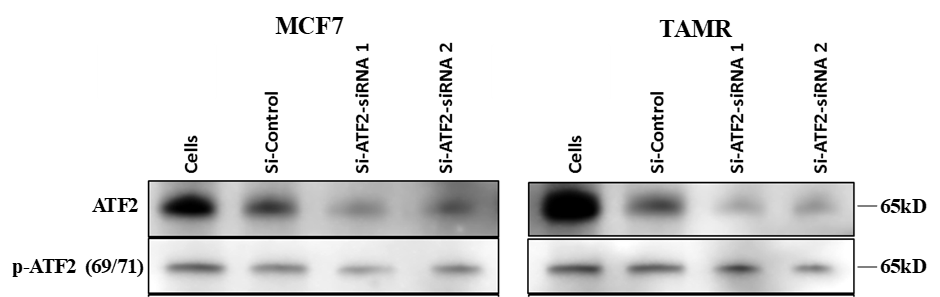


**Supplementary Figure 2**


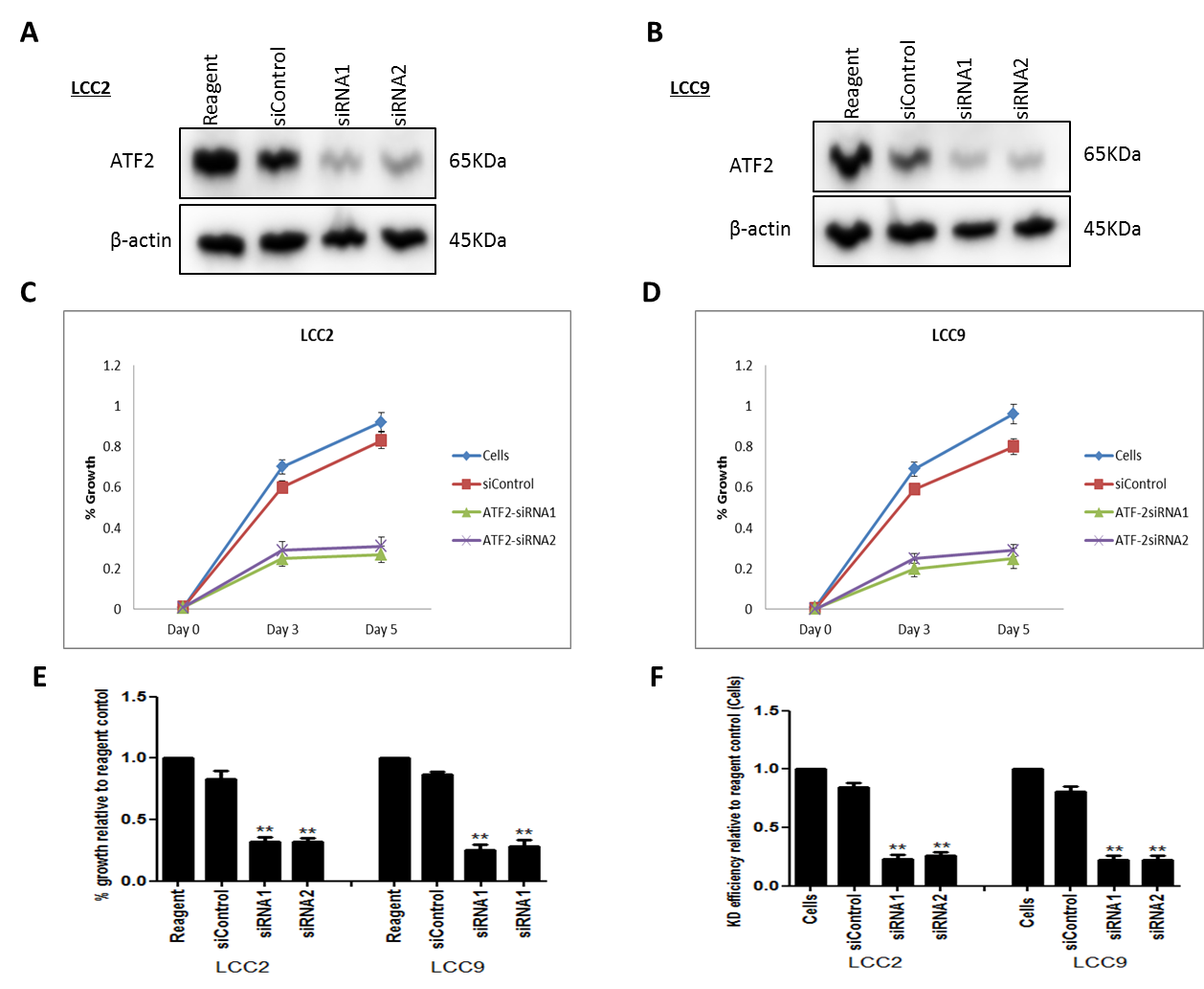


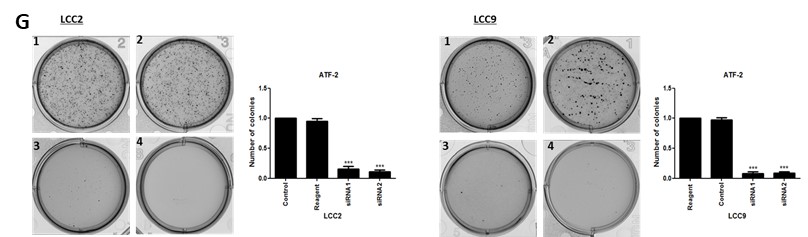


**Supplementary figure 3**


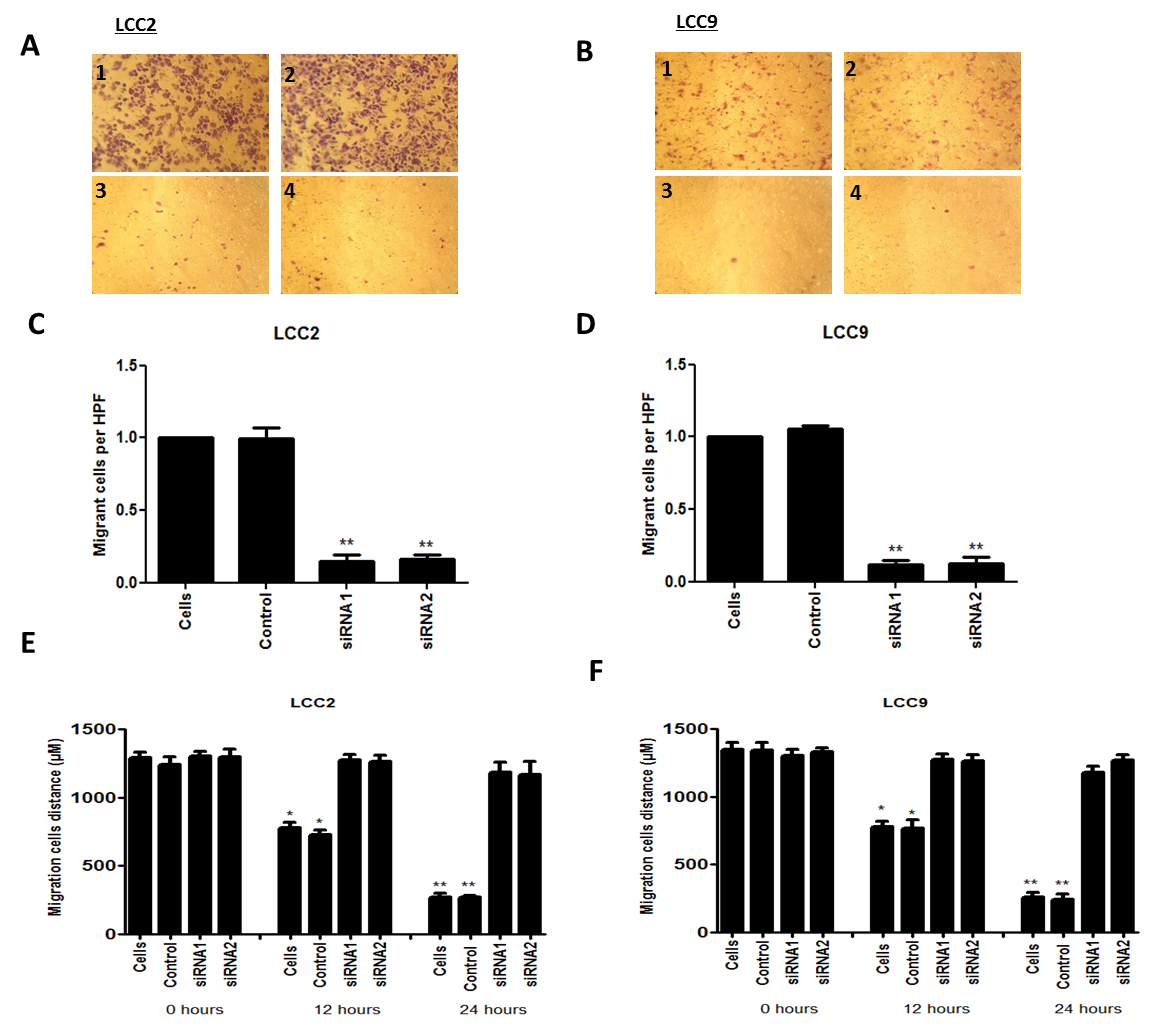


**Supplementary figure 4**

**
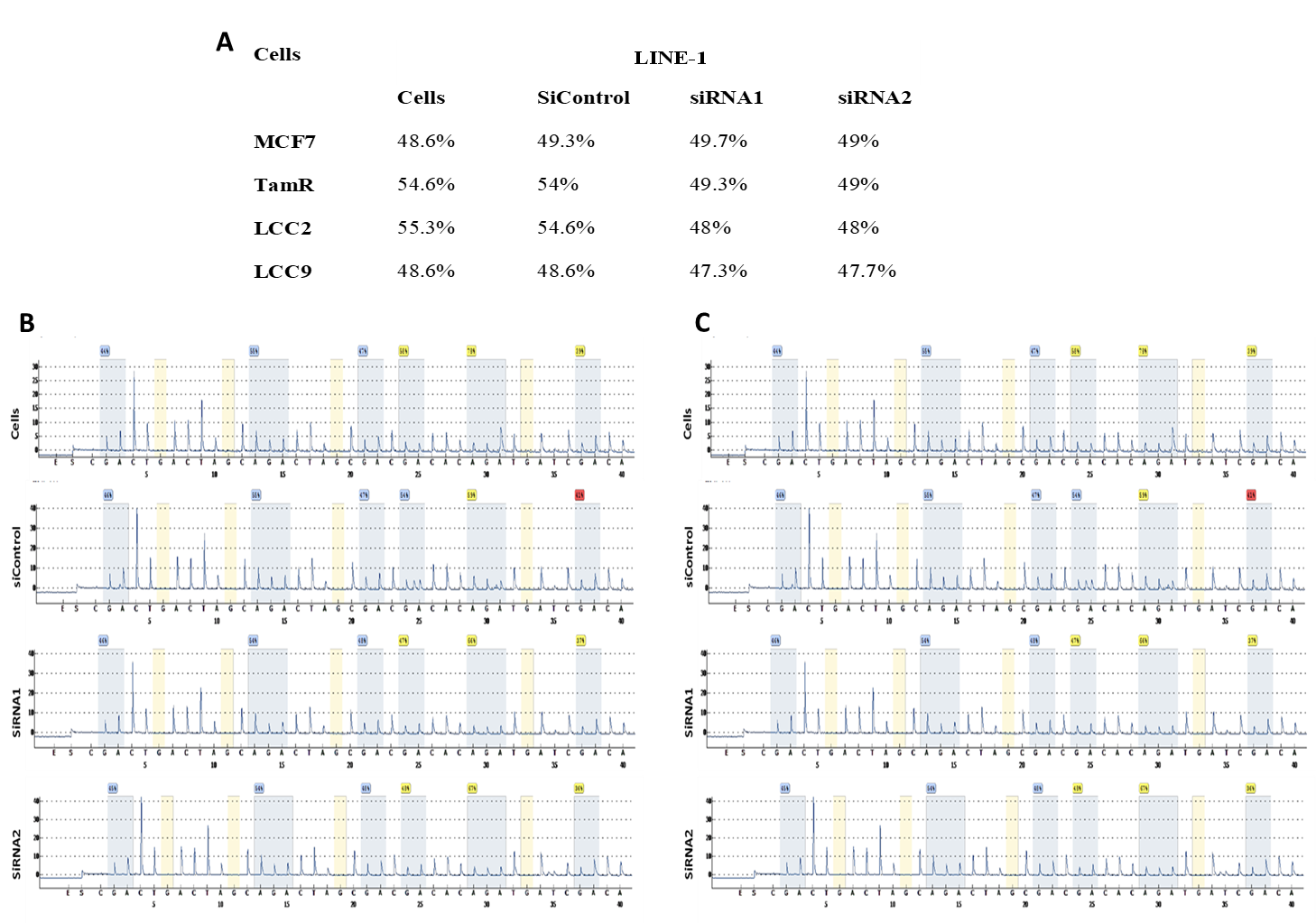
**

**Supplementary figure 5**


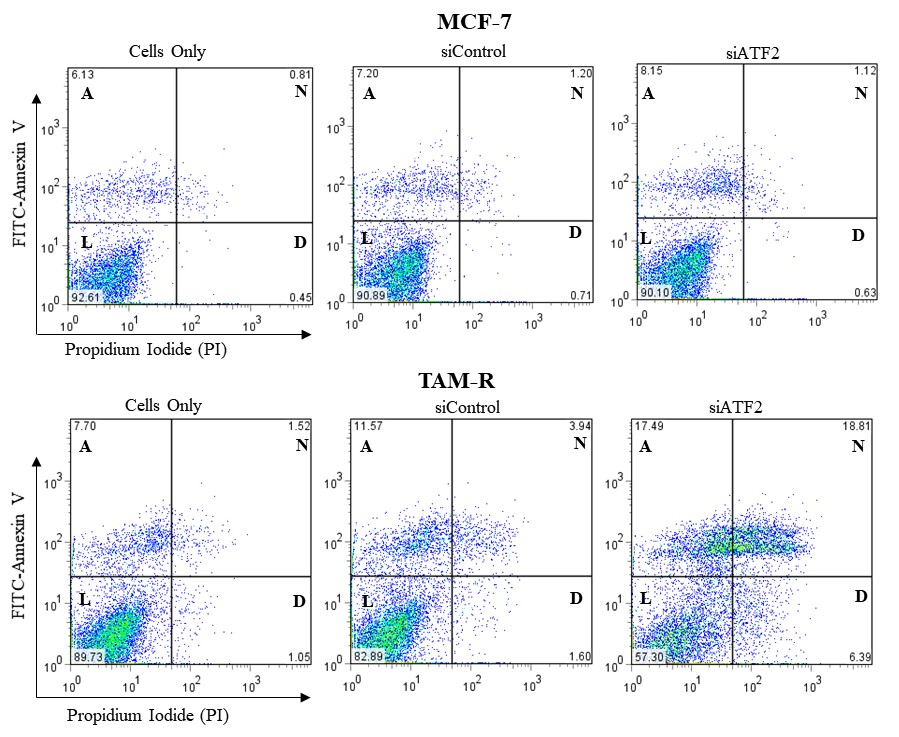


**Supplementary figure 6**

**
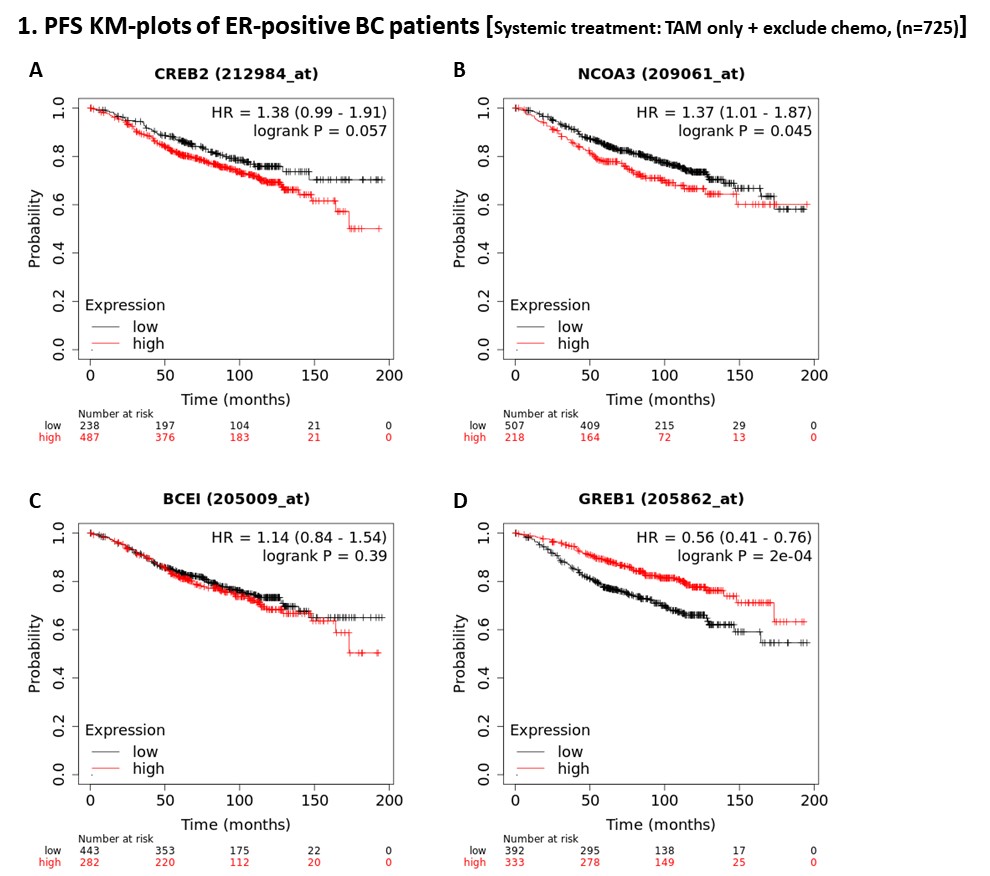
**

**
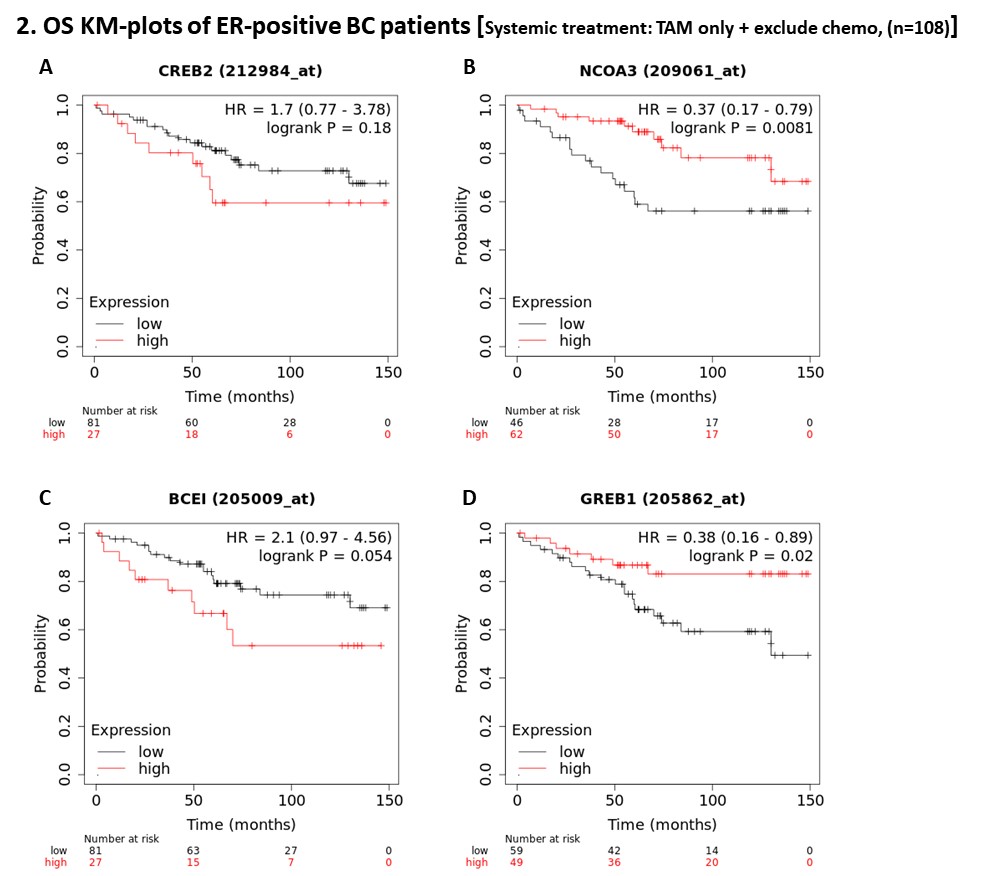
**
